# Colorectal cancer incidence and survival trends in Uganda: a population-based registry analysis (2000–20)

**DOI:** 10.1093/ije/dyag144

**Published:** 2026-08-11

**Authors:** Nicholas Matovu, Helen G Coleman, Gerald Mutungi, Michael Donnelly, Brian T Johnston, Maurice B Loughrey, Finian J Bannon, Charlene M McShane

**Affiliations:** Centre for Public Health, Queen’s University Belfast, Belfast, United Kingdom; Centre for Public Health, Queen’s University Belfast, Belfast, United Kingdom; Patrick G Johnston Centre for Cancer Research, Queen’s University Belfast, Belfast, United Kingdom; Department of Non-Communicable Diseases, Ministry of Health, Kampala, Uganda; Centre for Public Health, Queen’s University Belfast, Belfast, United Kingdom; Deartment of Gastroenterology, Belfast Health and Social Care Trust, Belfast, United Kingdom; Centre for Public Health, Queen’s University Belfast, Belfast, United Kingdom; Department of Cellular Pathology, Belfast Health and Social Care Trust, Belfast, United Kingdom; Centre for Public Health, Queen’s University Belfast, Belfast, United Kingdom; Centre for Public Health, Queen’s University Belfast, Belfast, United Kingdom

**Keywords:** colorectal cancer, incidence, trends, survival, Uganda, cancer epidemiology

## Abstract

**Introduction:**

The epidemiology of colorectal cancer (CRC) in Uganda is not sufficiently described, with the limited available data suggesting an increasing incidence. This study assessed CRC incidence and survival trends by using data from the two major population-based cancer registries in Uganda.

**Methods:**

We extracted incident CRC cases (ICD C18–C20) from the Kampala Cancer Registry (KCR; 2000–19) and the Gulu Cancer Registry (GCR; 2015–20). Crude and age-standardized incidence rates (ASIRs) and 1-, 3-, and 5-year survival estimates were calculated by using SEER*Stat software. Kaplan–Meier methods were applied to generate survival curves, with subgroup differences assessed by using log-rank tests. Joinpoint regression was used to evaluate trends in the incidence rates.

**Results:**

A total of 1118 CRC cases were identified. The mean age at diagnosis was 54.3 years (16.2 SD), with 37.8% diagnosed before age 50 years. The incidence was higher in the KCR compared with the GCR (crude: 2.4 vs. 0.8; ASIR: 9.2 vs. 2.0 per 100 000). A significant rise in ASIRs occurred exclusively among males in the KCR, with a 2.1% annual increase over 20 years (*P* < .001). In a sensitivity analysis restricted to histologically confirmed cases, the 1-, 3-, and 5-year relative survival in the KCR was 52.7%, 14.5%, and 4.3%, respectively.

**Conclusion:**

CRC incidence in Uganda remains low but is increasing among males. Four in 10 cases are diagnosed before age 50 years, suggesting a substantial burden of early-onset disease. Survival outcomes remain poor, stressing the need for strengthened prevention, early detection, and treatment services.

Key Messages

- We analysed population-based cancer registry data to understand the patterns of colorectal cancer incidence and survival in Uganda between 2000 and 2020.
- We observed a significant annual increase of 2.1% in incidence among men, along with a high burden of early-onset disease and very poor survival outcomes.
- These findings highlight urgent health system priorities for cancer prevention, timely diagnosis, and improved access to treatment.

## Introduction

GLOBOCAN data in 2022 estimated that Uganda had an age-standardized incidence rate (ASIR) of colorectal cancer (CRC) of 7.3 cases per 100 000 population [1]. While limited epidemiological data exist, regional differences in the CRC burden in Uganda are apparent. In an analysis of data from the Kampala Cancer Registry (KCR), Bukirwa *et al.* (2021) demonstrated a gradual increase in the incidence of CRC from 4.9 to 9.2 cases per 100 000 between 1991 and 2015, respectively [2]. A previous study that analysed data from the same registry between 1991 and 2010 also registered increasing CRC incidence for both males (from 7.8 to 8.8 per 100 000) and females (from 5.2 to 8.8 per 100 000) [3]. Data from the Northern region of Uganda, using a more recently established population-based Gulu Cancer Registry (GCR), reported a lower CRC incidence rate between 2013 and 2016 (males: 2.3 per 100 000; females: 1.3 per 100 000) [4] compared with that registered in the KCR. While a proportion of CRC can be attributed to underlying genetic predisposition [5], the rising incidence of colorectal tumours in Uganda is likely driven by changing lifestyle and dietary patterns [6]. These trends reflect a broader epidemiological transition in low- and middle-income countries toward lifestyle-related cancers. Moreover, the rising CRC rates are not unique to Uganda but are observed across sub-Saharan Africa, where all populations studied (including Congo, The Gambia, Mauritius, Kenya, Nigeria, etc.) in a population-based study showed increasing trends [7].

Other than the aforementioned studies, evidence on epidemiological and histopathological parameters for CRC in Uganda using population-based data is limited [8]. There is also a lack of survival data, with a population-based registry study published in 2021 using KCR data indicating a 5-year relative survival rate for CRC of 5.6% [9]. Studies using hospital data, although available, are also limited [10, 11].

Due to the gradually changing patterns and distribution of cancer risk factors in the Ugandan population [12], more up-to-date evidence on the broader CRC epidemiology using population-based data is warranted. The evidence from previous studies on CRC in Uganda is becoming outdated, with analyses extending only up to 2016 and lacking coverage across the country’s two major population-based cancer registries [2–4]. This limitation hinders comprehensive regional comparisons and obscures potential geographic variations in CRC epidemiology. Moreover, these studies have also not provided a detailed understanding of CRC incidence and survival trends, as stratification by tumour subsite remains absent. However, indications of an increase in CRC incidence warrant the need to monitor these trends, so as to provide evidence for timely policy and programme interventions. In this study, we update and expand the previous literature [2, 4, 9] by presenting a holistic picture of the CRC epidemiology in Uganda in which we analyse and present findings on the crude incidence rates and ASIRs, as well as trends in both incidence and survival. We also stratify results by key population subgroups and tumour subsites. This research will guide the planning and implementation of CRC prevention, early detection, and treatment initiatives in Uganda.

## Methods

### Data source and study population

We extracted data on all incident CRC cases using the International Classification of Diseases, Tenth Revision (ICD-10 codes C18–C20) from the KCR for 2000–19 and the GCR for 2015–20. Established in 1954, the KCR is located at the Department of Pathology, College of Health Sciences, Makerere University, Kampala district [13] and registers cancers within Kyadondo County [2]. The population of Kyadondo County was estimated at 3.4 million in 2014, representing ∼10% of the Ugandan population [14]. Previous studies report ∼90% completeness of KCR data [15], with morphological verification (MV) of 53.3% and death certificate only (DCO) cases of 3.1% [16], consistently with the acceptable data quality for similar settings [17].

The GCR, established in 2013, covers the Acholi subregion in Northern Uganda and is based at St. Mary’s Hospital Lacor in Gulu district [18]. It includes Gulu, Nwoya, Omoro, and Amuru districts, with a combined population of 762 343 in 2014 (2.2% of the national population) [19]. Although published indicators on completeness, MV%, and DCO% are limited, the GCR follows standardized population-based registration procedures guided by the International Agency for Research on Cancer and the African Cancer Registry Network (AFCRN) [20]. While GCR data were available from 2013, only data from 2015 to 2020 were included due to the absence of population denominators for earlier years.

Cases were assigned based on residence within registry catchment areas, minimizing duplication. The inclusion of both registries in this analysis enabled broader geographic representation and comparative analysis across distinct populations within Uganda, providing novel insights not addressed in prior studies. Ethical approval was obtained from the Makerere University School of Biomedical Sciences Research and Ethics Committee (SBS-2021–24) and the Uganda National Council of Science and Technology (HS1889ES), with administrative clearance from both registries.

### Variables extracted

Data extracted included demographic and clinical variables: patient ID, sex, tribe, age at diagnosis, date of birth, residence, basis of diagnosis, tumour stage, morphology, topography, behaviour, vital status, date of last contact, and ICD-10 code.

In both registries, some records had vital status and the date of last contact recorded at diagnosis without updates. In the KCR, registrars attempted follow-up through telephone contact with patients or next of kin. In the GCR, active follow-up was not feasible; therefore, the last recorded contact date was used for censoring. Survival time was calculated from date of diagnosis to last contact, death, or study end (31 December 2021).

### Data management and preparation

Data were reviewed for completeness and consistency before analysis. Datasets from both registries, initially in Excel format, were merged into a single dataset. The combined dataset was converted into .csv format and processed by using SEER*Prep software (v2.6.0, Information Management Services Inc., Calverton, MD), enabling compatibility with SEER*Stat following established procedures [21], which are further detailed in the Supplementary materials (Appendix 1).

### Statistical analysis

**Figure 1:**
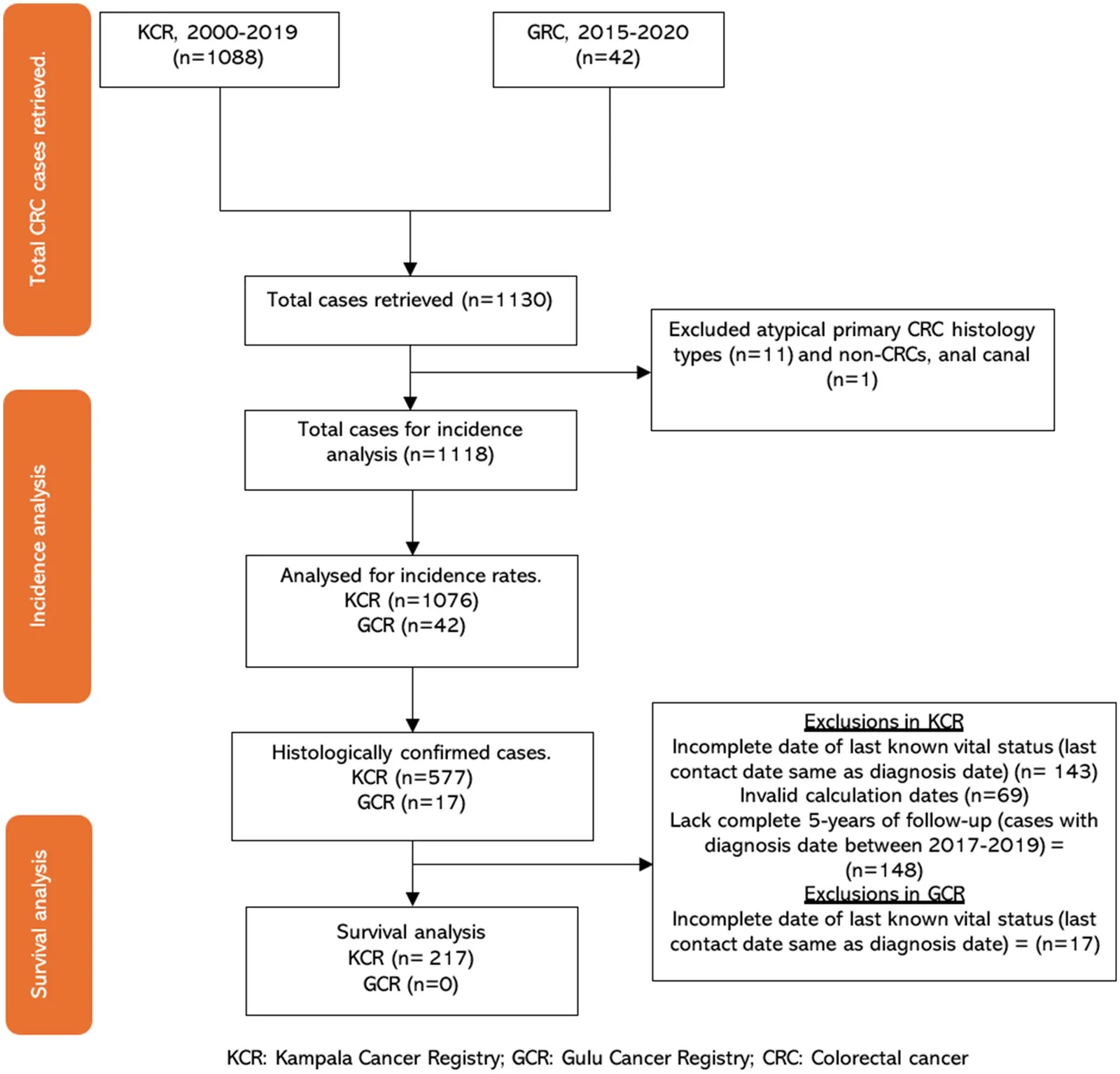
Flow diagram showing CRC cases considered in the different analyses conducted.

A flow diagram of CRC cases included in the analyses is shown in Fig. 1. SEER*Stat software (v8.3.9.2) was used to calculate crude, age-specific, and age-standardized incidence rates with corresponding confidence intervals (CIs). Incidence rates were calculated by using the number of CRC cases as the numerator and the respective catchment population as the denominator, expressed per 100 000 population. Population data were obtained from Uganda census sources [14]. ASIRs were computed by using direct standardization to the WHO standard population (2000–25) [22]. Incidence analyses were stratified by sex and anatomical subsite. Cases with atypical histology (*n* = 11) and those not colorectal in origin (anal canal; *n* = 1) were excluded.

Temporal trends in CRC incidence were assessed by using the Joinpoint Regression Program (version 4.8.0.1). This method fits log-linear models to ASIR data and identifies changes in temporal trends by using the Monte Carlo Permutation method [23]. A maximum of one joinpoint was allowed, enabling trend identification of up to two distinct time periods. Analyses were stratified by sex and anatomical subsite (colon: C18–C19; rectum: C20).

Survival analyses were conducted for both histologically confirmed cases [24] and the entire cohort. Observed survival (proportion of cancer cases alive after diagnosis) and relative survival (comparison with expected survival in the general population) were estimated at 1-, 3-, and 5-year intervals by using SEER*Stat, with corresponding confidence intervals (CIs). Accordingly, relative rather than net survival is reported, as it is more appropriate for population-based registry data in which the cause-of-death information is incomplete.

Analyses were stratified by sex, age group, tumour subsite, stage, and year of diagnosis. Observed survival was calculated as the proportion of patients alive at each time point, while relative survival was derived by dividing the observed survival by the expected survival. Expected survival was obtained from WHO Global Health Observatory life tables [25], which were expanded to single-year tables by using generalized linear models.

Case-listing survival data in SEER*Stat were exported to STATA 16 for further analysis. Kaplan–Meier survival curves were generated and survival differences between groups were assessed by using the log-rank test. This study adheres to the guidelines for reporting population-based cancer registry data (REPCAN) [26].

## Results

### Demographic, clinical, and epidemiological attributes of CRC cases retrieved across both registries

A total of 1118 CRC cases were identified across both registries. Overall, males and females were nearly equally represented. In the KCR, 1076 CRC cases were recorded over the 20-year period (2000–19), including 533 (49.5%) males and 543 (50.5%) females. In the GCR, 42 cases were recorded over 6 years (2015–20), comprising 27 (64.3%) males and 15 (35.7%) females (Table 1).

**Table 1:** Characteristics of CRC patients retrieved from the KCR (2000–19) and GCR (2015–20).

	Histologically confirmed CRC			All CRC cases		
Parameter	KCR (*n* = 577)	GCR (*n* = 17)	Overall (*n* = 594)	KCR (*n* = 1076)	GCR (*n* = 42)	Overall (*n* = 1118)
**Mean age (years) (±SD)**	52.73 (±16.17)	51.47 (±14.82)	52.69 (±16.12)	54.2 (±16.2)	56.7 (±15.8)	54.3 (±16.2)
**Age group (years)**						
** <50**	235 (40.7)	9 (52.9)	244 (41.1)	406 (37.7)	17 (40.5)	423 (37.8)
** 50–69**	241 (41.8)	5 (29.4)	246 (41.4)	456 (42.4)	15 (35.7)	471 (42.1)
** ≥70**	101 (17.5)	3 (17.6)	104 (17.5)	214 (19.9)	10 (23.8)	224 (20.1)
**Sex**						
** Male**	290 (50.3)	11 (64.7)	301 (50.7)	533 (49.5)	27 (64.3)	560 (50.1)
** Female**	287 (49.7)	6 (35.3)	293 (49.3)	543 (50.5)	15 (35.7)	558 (49.9)
**Anatomical subsite**						
** Colon**	288 (49.9)	9 (52.9)	297 (50.0)	563 (52.3)	25 (59.5)	588 (52.6)
** Rectum**	289 (50.1)	8 (47.1)	297 (50.0)	513 (47.7)	17 (40.5)	530 (47.4)
**Tumour stage**						
** Localized**	17 (2.9)	–	17 (2.9)	27 (2.5)	–	27 (2.4)
** Local spread**	43 (7.5)	–	43 (7.2)	48 (4.5)	–	48 (4.3)
** Regional spread**	47 (8.1)	–	47 (7.9)	58 (5.4)	–	58 (5.2)
** Advanced, metastatic**	27 (4.7)	1 (5.9)	28 (4.7)	37 (3.4)	1 (2.4)	38 (3.4)
** Unknown**	443 (76.8)	16 (94.1)	459 (77.3)	906 (84.2)	41 (97.6)	947 (84.7)
**Histological types**						
** Adenocarcinoma**	473 (82.0)	6 (35.3)	479 (80.6)	473 (44.0)	6 (14.3)	479 (42.8)
** Squamous cell carcinoma**	29 (5.0)	1 (5.9)	30 (5.1)	29 (2.7)	1 (2.4)	30 (2.7)
** Papillary adenocarcinoma**	24 (4.2)	0 (0.0)	24 (4.0)	24 (2.2)	0 (0.0)	24 (2.1)
** Carcinoma, NOS**	19 (3.3)	6 ((35.3)	25 (4.2)	19 (1.8)	6 (14.3)	25 (2.2)
** Mucinous adenocarcinoma**	11 (1.9)	0 (0.00)	11 (1.9)	11 (1.0)	0 (0.0)	11 (1.0)
** Malignant neoplasm, NOS**	0 (0.0)	0 (0.0)	0 (0.0)	499 (46.4)	25 (59.5)	524 (46.9)
** Others**	21 (3.6)	4 (23.5)	25 (4.2)	21 (2.0)	4 (9.5)	25 (2.2)

NOS, not otherwise specified; -, no cases were observed.

Across both registries, the mean age at diagnosis was 54.3 ± 16.2 years, with a high proportion of early-onset CRC (diagnosed before age 50 years) at 37.8%. There was a slight predominance of colonic cancers compared with rectal cancers (52.6% vs. 47.4%). The tumour stage was unknown for 84.7% (*n* = 947) of the cases. Among cases with staging data (*n* = 171), 56.1% (*n* = 96) had regionally advanced or metastatic disease, while 43.9% (*n* = 75) had localized or locally advanced disease (Table 1).

Of all CRC cases, 594 (53.1%) were histologically confirmed. Among these, 95.8% were carcinomas, predominantly adenocarcinomas (86.5%), followed by squamous cell carcinomas (5.1%) and carcinomas not otherwise specified (4.2%) (Table 1).

### Incidence of CRC in KCR and GCR

CRC incidence was higher in the KCR compared with the GCR. The crude incidence rate was 2.4 per 100 000 in the KCR and 0.8 per 100 000 in the GCR. Similarly, ASIRs were higher in the KCR (9.2 per 100 000) than in the GCR (2.0 per 100 000) (Table 2). Supplementary Figure S1a and b shows the ASIR trends over time for both registries.

**Table 2:** Trends in crude and age-standardized CRC incidence rates per 100 000 population, stratified by sex (KCR and GCR).

KCR [rate (95% CI)]	Estimate	2000–04	2005–09	2010–14	2015–19	Entire period 2000–19	APC (2000–19)
**Both sexes (*n* = 1076)**	Crude	2.1 (1.8, 2.4)	2.3 (2.0, 2.6)	2.6 (2.4, 3.0)	2.5 (2.2, 2.7)	2.4 (2.3, 2.5)	1.0 (−0.3, 2.3)
	ASIR	8.6 (7.2, 10.1)	8.7 (7.5, 10.0)	9.9 (8.7, 11.2)	9.6 (8.5, 10.8)	9.2 (8.6, 9.9)	0.6 (−0.7, 1.9)
**Males (*n* = 533)**	Crude	2.0 (1.5, 2.4)	2.2 (1.8, 2.7)	2.9 (2.5, 3.3)	2.7 (2.3, 3.2)	2.5 (2.3, 2.7)	2.2 (0.4, 4.0)^a^
	ASIR	7.8 (6.0, 10.1)	8.3 (6.5, 10.2)	10.9 (9.1, 12.9)	10.8 (9.0, 12.8)	9.7 (8.7, 10.7)	2.1 (0.1, 4.1)^a^
**Females (*n* = 543)**	Crude	2.3 (1.8, 2.7)	2.3 (1.9, 2.7)	2.5 (2.1, 2.9)	2.2 (1.9, 2.6)	2.3 (2.1, 2.5)	0.0 (−1.4, 1.5)
	ASIR	9.3 (7.4, 11.4)	9.0 (7.3, 10.9)	9.2 (7.6, 10.9)	8.6 (7.2, 10.2)	8.9 (8.1, 9.7)	−0.6 (−1.9, 0.8)
**GCR [rate (95% CI)]**					**2015–19**	**Entire period 2015–20**	**APC (2015–20)**
**Both sexes (*n* = 42)**	Crude				0.9 (0.6, 1.2)	0.8 (0.6, 1.1)	2.3 (−17.8, 27.3)
	ASIR				2.1 (1.5, 2.9)	2.0 (1.4, 2.7)	5.5 (−17.2, 34.5)
**Males (*n* = 27)**	Crude				1.2 (0.8, 1.8)	1.1 (0.7, 1.5)	−5.6 (−29.7, 26.8)
	ASIR				3.2 (2.0, 4.8)	2.8 (1.8, 4.2)	−4.1 (−32.0, 35.2)
**Females^b^ (*n* = 15)**	Crude				0.6 (0.3, 1.0)	0.6 (0.3, 0.9)	–
	ASIR				1.3 (0.7, 2.2)	1.3 (0.7, 2.1)	–

a Significant changes in CRC incidence observed during the given period, *P* < .05.

b APCs for females in the GCR could not be calculated due to zero cases recorded across some years (2015–18).

APC, annual percentage change.

### Trends in CRC incidence in KCR and GCR, by sex

**Figure 2:**
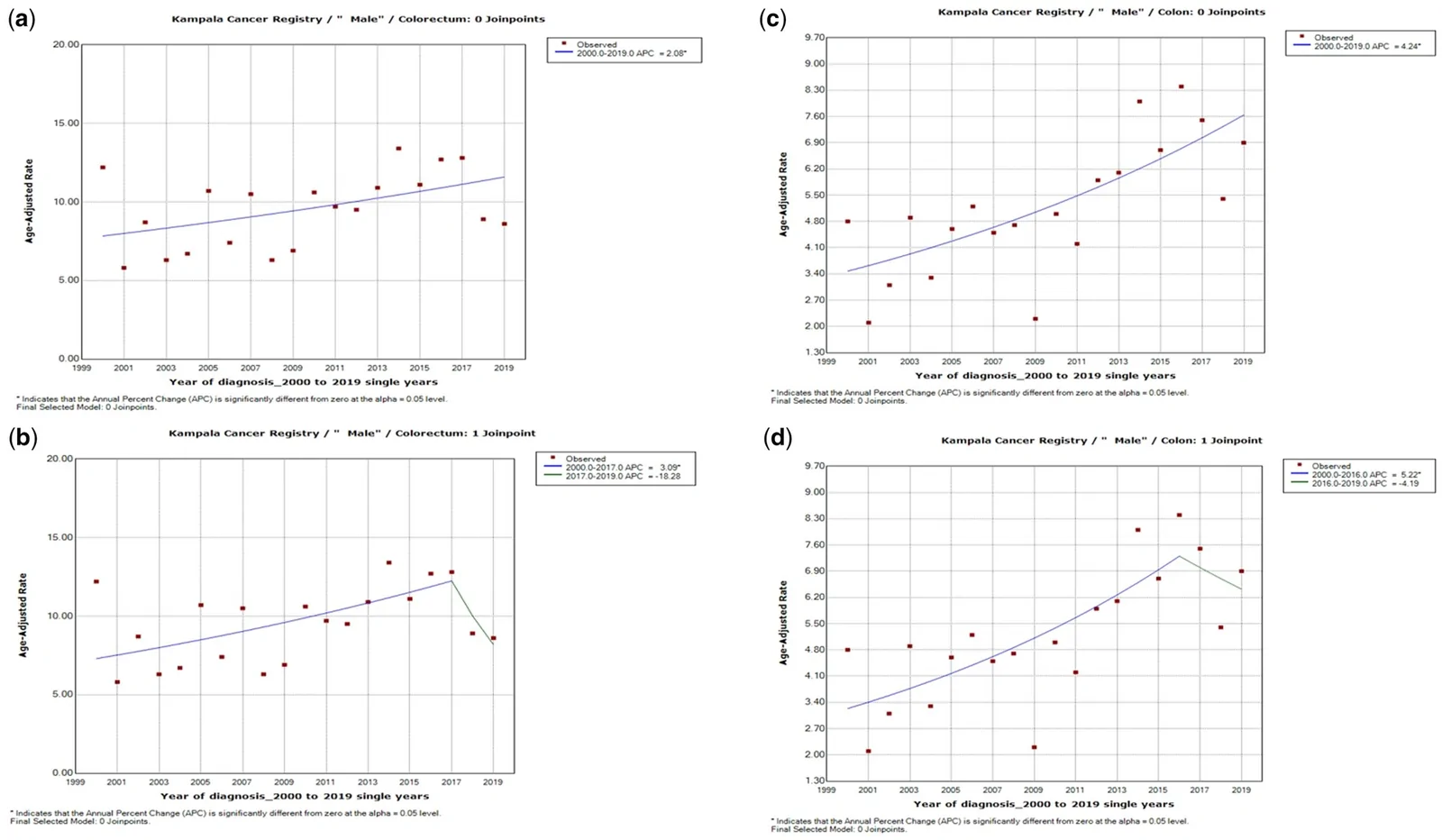
Joinpoint regression for trends in (a) CRC (males, 0 joinpoints), (b) CRC (males, 1 joinpoint), (c) colon cancer (males, 0 joinpoints), and (d) colon cancer (males, 1 joinpoint).

Among males in the KCR, the CRC incidence increased by an average of 2.1% annually over the entire 20-year period (zero joinpoints) [annual percentage change (APC): 2.1%; 95% CI: 0.1%, 4.1%; *P* < .001] (Table 2 and Fig. 2a). When one joinpoint was allowed, two distinct trends were identified, with an inflection point in 2017. CRC incidence increased by 3.1% annually from 2000 to 2017 (APC: 3.1%; 95% CI: 0.7%, 5.5%, *P* < .001) followed by an annual decrease of 18.3% (95% CI: −55.5, 50.2) from 2017 to 2019 (Fig. 2b).

In the GCR, there was little evidence of temporal changes in crude or age-standardized incidence rates for either sex during the study period (Table 2).

### Trends in CRC incidence in KCR and GCR, by anatomical subsite and sex

Among males in the KCR, colonic cancer incidence increased by an average of 4.2% annually over the full study period (APC: 4.2%; 95% CI: 2.1%, 6.4%; *P* < .001) (Table 3 and Fig. 2c). With one joinpoint, two phases were identified, with an inflection in 2016: an annual increase of 5.2% from 2000 to 2016 (95% CI: 2.2%, 8.4%, *P* < .001) followed by a decrease of 4.2% (95% CI: −27.0, 25.8) from 2016 to 2019 (Fig. 2d).

**Table 3:** Trends in crude and age-standardized colon and rectal cancer incidence rates, stratified by sex (KCR and GCR).

KCR [rate (95% CI)]	Estimate	2000–04	2005–09	2010–14	2015–19	Entire period 2000–19	APC (2000–19)
**Colon, both sexes (*n* = 563)**	Crude	1.0 (0.8, 1.2)	1.0 (0.8, 1.2)	1.4 (1.2, 1.6)	1.4 (1.2, 1.4)	1.3 (1.2, 1.4)	2.6 (0.7, 4.6)**^a^**
	ASIR	4.3 (3.3, 5.4)	4.1 (3.3, 5.1)	5.3 (4.4, 6.2)	5.9 (5.0, 6.9)	5.0 (4.5, 5.5)	2.4 (0.5, 4.2)**^a^**
**Colon, males (*n* = 291)**	Crude	0.9 (0.6, 1.2)	1.1 (0.8, 1.4)	1.6 (1.3, 2.0)	1.7 (1.4, 2.0)	1.4 (1.2, 1.5)	4.0 (1.5, 6.6)**^a^**
	ASIR	3.7 (2.4, 5.3)	4.2 (3.0, 5.6)	5.9 (4.6, 7.4)	6.9 (5.5, 8.6)	5.4 (4.7, 6.2)	4.2 (2.1, 6.4)**^a^**
**Colon, females (*n* = 272)**	Crude	1.1 (0.8, 1.5)	1.0 (0.8, 1.3)	1.2 (1.0, 1.5)	1.2 (1.0, 1.5)	1.2 (1.0, 1.3)	1.0 (−1.1, 3.0)
	ASIR	4.8 (3.5, 6.5)	4.0 (2.9, 5.4)	4.8 (3.7, 6.1)	4.9 (3.9, 6.2)	4.6 (4.1, 5.3)	0.5 (−1.7, 2.7)
**Rectum, both sexes (*n* = 513)**	Crude	1.1 (0.9, 1.4)	1.2 (1.0, 1.5)	1.2 (1.0, 1.4)	1.0 (0.9, 1.2)	1.1 (1.0, 1.2)	−0.7 (−2.2, 0.9)
	ASIR	4.3 (3.4, 5.4)	4.6 (3.7, 5.5)	4.6 (3.8, 5.5)	3.7 (3.1, 4.5)	4.2 (3.8, 4.7)	−1.5 (−3.4, 0.5)
**Rectum, males (*n* = 242)**	Crude	1.1 (0.8, 1.4)	1.2 (0.9, 1.5)	1.3 (1.0, 1.6)	1.1 (0.8, 1.3)	1.1 (1.0, 1.3)	−0.4 (−2.5, 1.7)
	ASIR	4.2 (2.9, 5.8)	4.1 (2.9, 5.5)	5.0 (3.8, 6.4)	3.8 (2.8, 5.0)	4.3 (3.7, 4.9)	−1.1 (−4.5, 2.5)
**Rectum, females (*n* = 271)**	Crude	1.1 (0.8, 1.5)	1.3 (1.0, 1.6)	1.2 (1.0, 1.5)	1.0 (0.8, 1.3)	1.1 (1.0, 1.3)	−0.7 (−2.8, 1.4)
	ASIR	4.4 (3.1, 6.0)	5.0 (3.8, 6.4)	4.4 (3.3, 5.6)	3.7 (2.8, 4.7)	4.2 (3.7, 4.8)	−1.6 (−3.6, 0.5)
**GCR [rate (95% CI)]**					**2015–19**	**Entire period 2015–20**	**APC (2015–20)**
**Colon, both sexes**	Crude				0.5 (0.3, 0.8)	0.5 (0.3, 0.7)	**−**4.7 (−23.4, 18.7)
	ASIR				1.2 (0.7, 1.8)	1.1 (0.7, 1.6)	−7.7 (−29.2, 20.4)
**Colon, males (*n* = 14)**	Crude				0.6 (0.3, 1.1)	0.5 (0.3, 0.9)	−9.1 (−30.6, 19.2)
	ASIR				1.5 (0.8, 2.6)	1.3 (0.7, 2.2)	−14.4 (−35.7, 13.9)
**Colon, females^b^ (*n* = 11)**	Crude				0.4 (0.2, 0.8)	0.4 (0.2, 0.7)	–
	ASIR				0.9 (0.4, 1.8)	0.9 (0.4, 1.6)	–
**Rectum, both sexes**	Crude				0.4 (0.2, 0.6)	0.3 (0.2, 0.5)	12.2 (−26.4, 71.1)
	ASIR				0.9 (0.5, 1.5)	0.9 (0.5, 1.4)	20.5 (−24.5, 92.2)
**Rectum, males (*n* = 13)**	Crude				0.6 (0.3, 1.0)	0.5 (0.3, 0.9)	−0.8 (−41.9, 69.4)
	ASIR				1.7 (0.9, 2.9)	1.5 (0.8, 2.6)	5.1 (−43.5, 95.8)
**Rectum, females^b^ (*n* = 4)**	Crude				0.1 (0.0, 0.4)	0.2 (0.0, 0.4)	–
	ASIR				0.3 (0.1, 0.9)	0.4 (0.1, 0.9)	–

a Significant changes in CRC incidence observed during the given period, *P* < .05;

b APCs for females in the GCR could not be calculated due to zero cases recorded across some years (2015–18).

Among females in the KCR, there was little evidence of temporal changes in colonic cancer incidence, and rectal cancer incidence showed little evidence of change in either sex (Supplementary Figure 2a–c). Similarly, there was little evidence of temporal changes in colonic or rectal cancer incidence in the GCR (Table 3).

### Age-specific incidence rates for CRC in KCR and GCR

**Figure 3:**
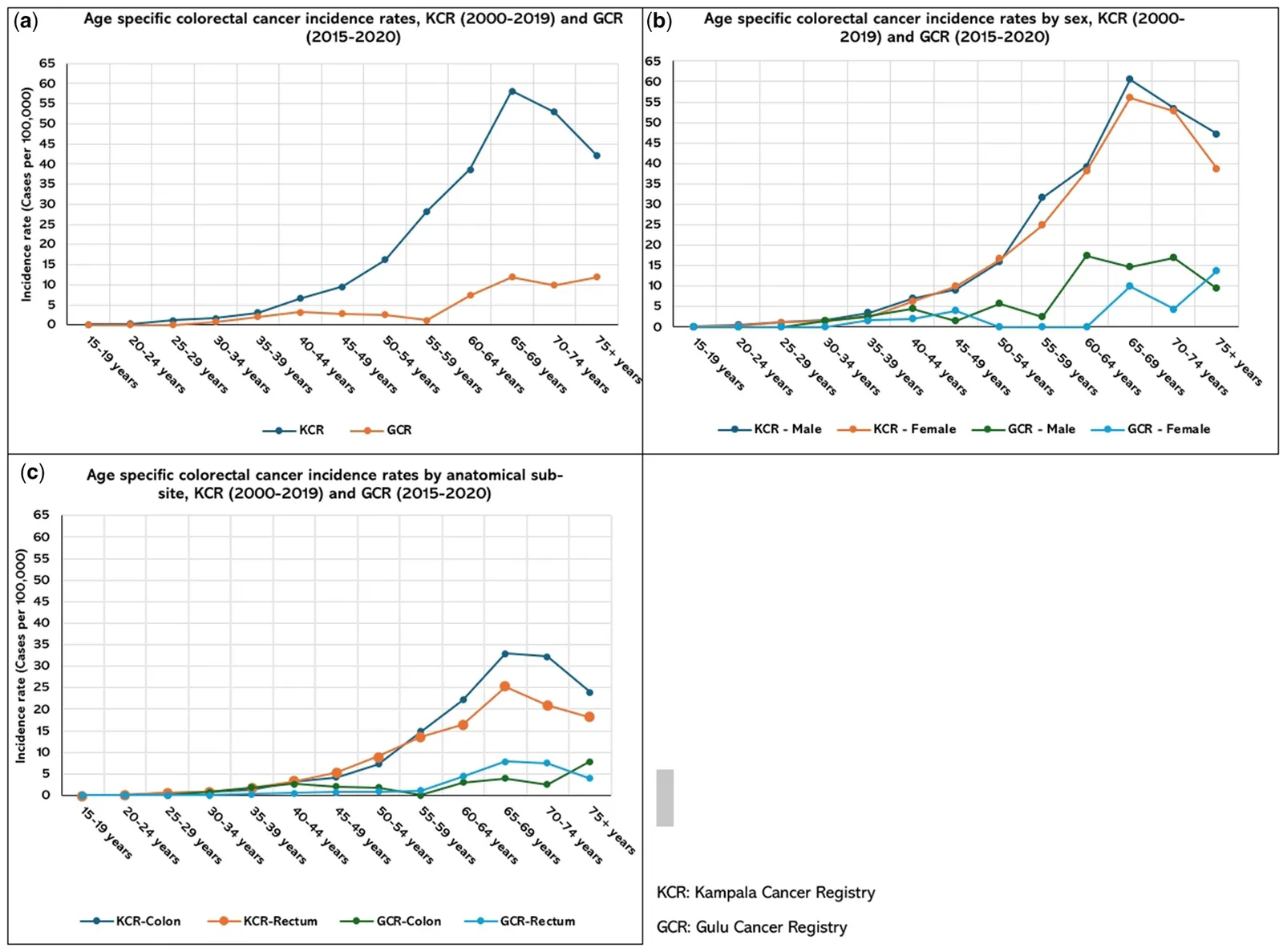
Age-specific incidence rates for CRC in KCR and GCR. (a) Overall stratified by registry, (b) stratified by sex and registry, and (c) stratified by tumour subsite and registry.

Age-specific CRC incidence rates were consistently higher in the KCR compared with the GCR from age 20 years onwards (Fig. 3a). In the KCR, the incidence increased gradually from ages 20–49 years, followed by a steeper rise, peaking at 58.1 per 100 000 in the age group of 65–69 years before declining in older ages. In the GCR, the incidence increased more gradually with age, peaking at 12 cases per 100 000 in the age group of 65–69 years.

Sex-stratified analyses showed similar patterns between males and females in the KCR (Fig. 3b). In the GCR, the incidence increased gradually, with a slightly younger peak among males (60–64 years) compared with females (≥75  years).

By anatomical subsite (Fig. 3c), colonic and rectal cancer incidence rates were similar up to ages 55–59 years in both registries. At older ages, the colonic cancer incidence exceeded the rectal cancer incidence in the KCR, while the opposite pattern was observed in the GCR, although data for those aged ≥75 years also showed higher colonic cancer incidence in the GCR.

### Survival rates of CRC patients

Survival analysis included 217 histologically confirmed CRC cases, of which 124 (57.1%) were males and 93 (42.9%) were females. The mean follow-up time was 11.8 months (13.6 SD; range: 1–60 months). No cases from the GCR were included due to complete loss to follow-up.

The overall observed survival was 51.3% (95% CI: 42.9%, 59.1%) at 1 year, 13.4% (95% CI: 8.1%, 20.1%) at 3 years, and 3.7% (95% CI: 1.1%, 9.2%) at 5 years (Table 4). Survival estimates were broadly comparable across subgroups defined by sex, tumour site, stage, age, and year of diagnosis (Supplementary Figure 3a–e).

**Table 4:** Observed survival of CRC patients (*n* = 217), KCR (2000–16).

Subgroup	Year 1				Years 2 and 3				Years 4 and 5				MOST (months)
	Alive at start [*n* (%)]	Deaths [*n* (%)]	LTFU [*n* (%)]	OS, 1 year [% (95% CI)]	Alive at start [*n* (%)]	Deaths [*n* (%)]	LTFU [*n* (%)]	OS, 3 years [% (95% CI)]	Alive at start [*n* (%)]	Deaths [*n* (%)]	LTFU [*n* (%)]	OS, 5 years [% (95% CI)]	
**Overall**	217 (100)	72 (33.2)	74 (34.1)	51.3 (42.9, 59.1)	71 (32.7)	48 (22.1)	9 (4.1)	13.4 (8.1, 20.1)	14 (6.5)	9 (4.1)	3 (1.4)	3.7 (1.1, 9.2)	13.2
**Sex**													
** Male**	124 (100)	39 (31.5)	42 (33.9)	54.7 (43.4, 64.6)	43 (34.7)	30 (24.2)	3 (2.4)	14.2 (7.4, 23.1)	10 (8.1)	6 (4.8)	3 (2.4)	4.7 (1.2, 12.0)	13.9
** Female**	93 (100)	33 (35.5)	32 (34.4)	46.8 (33.9, 58.7)	28 (30.1)	18 (19.4)	6 (6.5)	12.6 (5.2, 23.5)	4 (4.3)	3 (3.2)	0 (0.0)	3.1 (0.3, 13.2)	10.6
**Tumour subsite**													
** Colon**	106 (100)	31 (29.2)	36 (34.0)	57.5 (45.2, 68.0)	39 (36.8)	28 (26.4)	3 (2.8)	13.9 (6.9, 23.5)	8 (7.5)	4 (3.8)	3 (2.8)	5.0 (1.0, 14.6)	16.2
** Rectum**	111 (100)	41 (36.9)	38 (34.2)	45.4 (33.8, 56.3)	32 (28.8)	20 (18.0)	6 (5.4)	13.9 (6.6, 23.7)	6 (5.4)	5 (4.5)	0 (0.0)	2.3 (0.2, 10.3)	10.4
**Stage**													
** I and II**	32 (100)	12 (37.5)	5 (15.6)	55.7 (35.3, 71.9)	15 (46.9)	10 (31.3)	1 (3.1)	17.8 (6.1, 34.4)	4 (12.5)	4 (12.5)	0 (0.0)	0.0	13.8
** III and IV**	35 (100)	17 (48.6)	4 (11.4)	45.6 (27.7, 61.8)	14 (40.0)	11 (31.4)	2 (5.7)	7.8 (1.5, 21.5)	1 (2.9)	0 (0.0)	1 (2.9)	−^a^	10.6
** Unknown**	150 (100)	43 (28.5)	65 (43.0)	52.7 (41.8, 62.5)	42 (28.0)	27 (18.0)	6 (4.0)	13.8 (7.0, 22.9)	9 (6.0)	5 (3.3)	2 (1.3)	6.1 (2.0, 13.6)	13.6
**Age at diagnosis (years)**													
** <50**	91 (100)	37 (40.7)	25 (27.5)	46.0 (33.8, 57.4)	29 (31.9)	21 (23.1)	3 (3.3)	9.2 (3.4, 18.4)	5 (5.5)	3 (3.3)	1 (1.1)	2.8 (0.3, 11.1)	10.2
** 50–69**	90 (100)	25 (27.8)	37 (41.1)	54.2 (39.9, 66.4)	28 (31.1)	16 (17.8)	5 (5.6)	20.1 (10.0, 32.6)	7 (7.8)	4 (4.4)	2 (2.2)	8.2 (2.1, 19.8)	13.6
** ≥70**	36 (100)	10 (27.0)	12 (32.4)	60.7 (39.3, 76.6)	14 (38.9)	11 (32.4)	1 (2.7)	12.1 (3.1, 27.9)	2 (5.6)	2 (5.6)	0 (0.0)	0.0	14.7
**Year of diagnosis**													
** 2000–10**	62 (100)	26 (41.9)	19 (30.6)	41.8 (27.0, 56.0)	17 (27.4)	9 (14.5)	3 (4.8)	15.8 (6.1, 29.5)	5 (8.1)	4 (6.5)	0 (0.0)	3.2 (0.2, 13.7)	9.9
** 2011–16**	155 (100)	46 (29.5)	55 (35.3)	55.7 (45.6, 64.7)	54 (34.8)	39 (25.2)	6 (3.8)	12.5 (6.6, 20.3)	9 (5.8)	5 (3.2)	3 (1.9)	4.0 (0.8, 11.8)	14.4

a Last patient was censored before the end of the period, at 3.6 years.

OS, observed survival; LTFU, lost to follow-up; MOST, median observed survival time.

**Figure 4:**
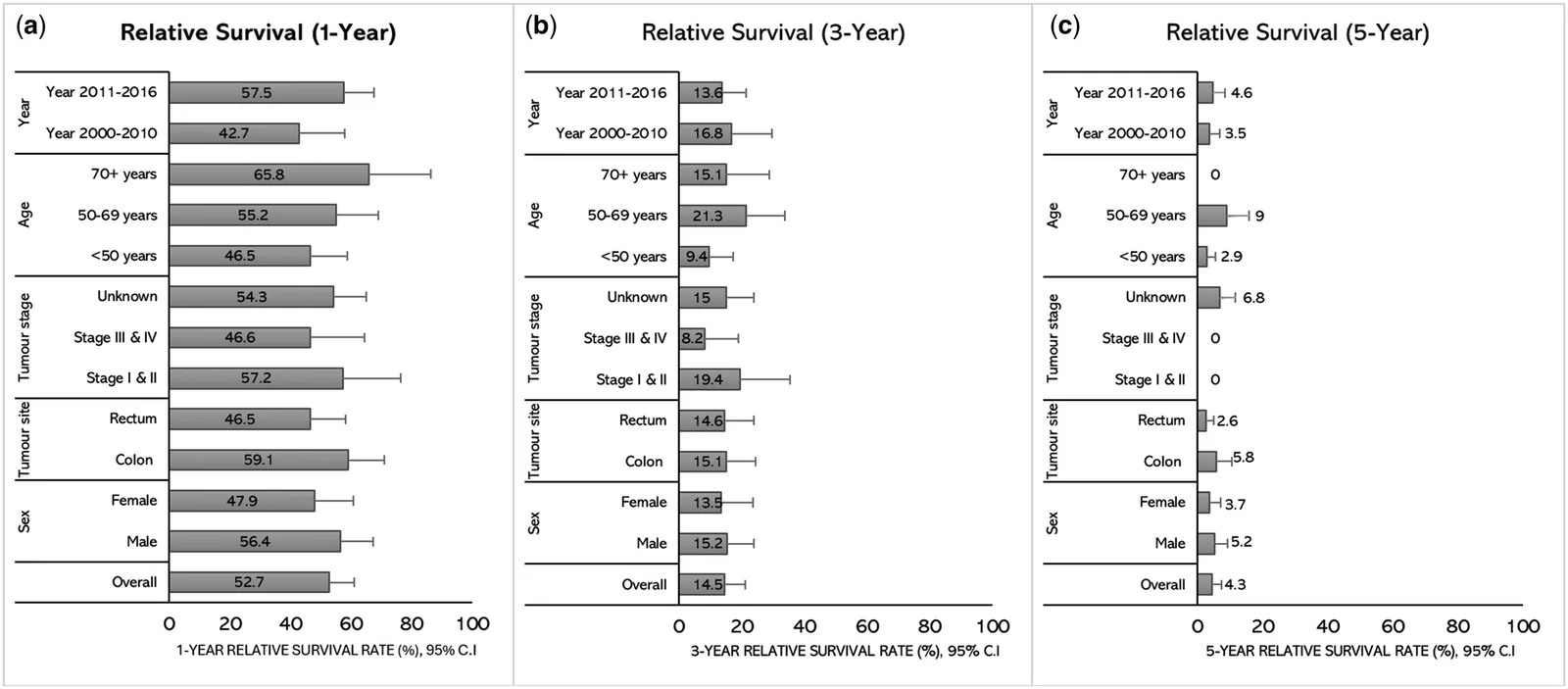
Relative survival rates of CRC in the KCR (2000–16), stratified by sex, tumour site, tumour stage, age, and year of diagnosis. (a) One-year relative survival rates, (b) 3-year relative survival rates, and (c) 5-year relative survival rates.

The relative survival was 52.7% (95% CI: 44.0%, 60.7%) at 1 year, 14.5% (95% CI: 8.7%, 21.6%) at 3 years, and 4.3% (95% CI: 1.2%, 8.5%) at 5 years (Fig. 4). Relative survival was higher among males and patients with colonic tumours compared with their respective counterparts across all time points. Survival estimates from the entire cohort were largely consistent with those observed in the histologically confirmed cohort (Supplementary Table S1 and Figs 4 and 5).

## Discussion

In this study using data from two population-based cancer registries in Uganda, the ASIR of CRC ranged from 2.0 to 9.2 per 100 000 in the GCR and KCR, respectively. The CRC incidence among males in the KCR increased by an average of 2.1% annually. Survival analysis of histologically confirmed cases revealed low relative survival at 1, 3, and 5 years (52.7%, 14.5%, and 4.3%, respectively).

Although the CRC incidence in Uganda remains lower than those in Western countries [27], CRC incidence among males in the KCR increased over the 20-year study period. Previous work using KCR data reported increasing incidence in both sexes between 1991 and 2015 (APC males: 2.2%, females: 1.7%) [2]. The lack of evidence for a temporal change among females in the current study may reflect differences in study periods, as more recent data (2000–19) likely capture a period of peak incidence among females [3]. Across Africa, the CRC ASIRs increased by 11% annually between 2010 and 2019 [28]. These increases may reflect epidemiological transitions associated with urbanization, Westernized diets, alcohol consumption, and reduced physical activity [29], all of which are established CRC risk factors [30]. However, more research is needed to understand other factors (e.g. healthcare/health system or registry processes) that may influence the observed incidence changes in our study.

The higher CRC incidence observed in the KCR compared with the GCR may reflect urban–rural differences. The GCR population is predominantly rural, with presumably limited diagnostic capacity and healthcare access, and potentially lower exposure to risk factors such as processed foods and alcohol. Urban residence has been associated with increased CRC risk in Uganda [6] and elsewhere in Africa [31]. The lack of evidence for temporal incidence trends in the GCR may also reflect the relatively short observation period (2015–20). Additionally, the joinpoint analyses in both registries may reflect random variation rather than true epidemiological changes.

Age-specific incidence rates peaked at 65–69 years in both registries, consistently with findings from Zambia [32] and within the broader African range of 50–69 years [33–36]. In contrast, the peak CRC incidence occurs at older ages (70–80 years) in other middle- to high-income settings such as China [37], the USA [38], and Europe [39, 40], reflecting later age at diagnosis in these regions compared with Uganda or even Africa at large [41]. Our findings also show a high proportion (37.8%) of CRC cases diagnosed before age 50 years, though this should be interpreted in the context of Africa’s younger population structure. While early-onset CRC in African populations may reflect both hereditary and sporadic disease [42], the overall incidence in younger populations age <50 years remains low (i.e. <12 per 100 000) [41]. These findings may inform decisions on CRC screening initiation in Uganda and similar settings.

Very few studies have examined CRC survival rates in Uganda, or indeed in Africa more broadly. CRC survival in Uganda remains poor. The observed and relative survival estimates in this study were comparable to those reported by Gullickson *et al.* [9]. The 5-year relative survival in Uganda appears lower than those in other African settings, including Ethiopia (23%), Zimbabwe (39%), and Côte d’Ivoire (41%) [9]. This may indicate challenges related to late presentation, limited treatment availability, and access to care. Supporting this, a study at the Uganda Cancer Institute reported delays in treatment initiation among 34% of cancer patients, often due to financial constraints [43]. Such barriers likely contribute to poor prognosis and highlight the need for improved cancer care systems.

### Study strengths and limitations

A major strength of this study is the use of recent data from Uganda’s two principal population-based cancer registries, enabling regional comparisons and improving representativeness. The study also aligns with AFCRN priorities by highlighting the need to strengthen cancer surveillance across Africa [44].

However, several limitations should be noted. Survival analyses were affected by incomplete vital status data, with some patients lost to follow-up despite attempts to trace them via telephone. The small sample size in the GCR (42 cases over 6 years) limited statistical power, restricted subgroup analyses, and resulted in wide CIs. Additionally, all GCR cases were excluded from survival analysis due to complete loss to follow-up, reducing the national representativeness of survival estimates. Enhancing case follow-up would improve data quality and analytical power in future studies. Notwithstanding these limitations, the inclusion of GCR data was important for capturing regional diversity and enabling preliminary comparisons across Uganda.

Finally, a key limitation was the high proportion of cases with unknown tumour stage, which constrained stage-specific analyses and may have introduced uncertainty in the interpretation of stage-related findings; these results should therefore be interpreted with caution. Missing staging data in African cancer registries is largely attributed to limited diagnostic capacity, incomplete clinical documentation, fragmented data systems, and resource constraints [45, 46]. Variability in staging systems and incomplete case ascertainment further contribute to missing stage data [47, 48]. Addressing these challenges is essential to improve data completeness and strengthen cancer surveillance systems in Uganda and across Africa [49].

## Conclusion

This population-based registry study provides an updated profile of the epidemiology of CRC in Uganda. The incidence of CRC, although still relatively low, has seen gradual increases over the past two decades, most especially in colonic cancers among males. The 5-year CRC relative survival rates remain very low in this population. These findings highlight the need to strengthen population-based cancer registries to improve surveillance, alongside expanding prevention, early detection, and treatment services to improve CRC outcomes in Uganda.

## Ethics approval

Ethical approval was obtained from the Makerere University School of Biomedical Sciences Research and Ethics Committee (SBS-2021–24) and the Uganda National Council of Science and Technology (HS1889ES), with administrative clearances obtained from both registries.

## Supplementary Material

dyag144_Supplementary_Data

## Data Availability

The data underlying this article were provided by the Kampala Cancer Registry and Gulu Cancer Registry with permission. Data will be shared on request to the corresponding author with permission from both registries.
