# Supplementary material for: Colorectal cancer incidence and survival trends in Uganda: a population-based registry analysis (2000–20)
Source: Int J Epidemiol. 2026 Aug 11;55(4):dyag144. doi: 10.1093/ije/dyag144 (PMC13461188; doi:10.1093/ije/dyag144)
Supplement: Supplementary file 1 [file dyag144_supplementary_data.docx]

**Appendix 1: Data preparation for incidence calculations in SEER*Stat, Brief description:**

Briefly, original database description (dd) files provided within the SEER*Prep software were customised to fit the variables available within the local cancer registry dataset i.e. the combined KCR and GCR dataset. Using Fixlen software version 1.8, the combined csv format dataset from both KCR and GCR was converted to the fixed length format (.txd) to create a “cases file” for the incidence database. A similar file (in the csv format) containing data on the respective population coverage (by sex) of KCR and GCR was created and converted to the fixed length format (.txd) to create a “population file” for the incidence database. Both the cases and population files were imported into the SEER*Prep software to create an incidence database. The created incidence database was then imported into the SEER*Stat software for analysis.

**Supplementary figures and tables:**

a

b

Colon

ASIR (cases per 100,000)

| Years | KCR Colon (Male) | KCR Colon (Female) | GCR Colon (Male) | GCR Colon (Female) |
| --- | --- | --- | --- | --- |
| 2000 | 4.8 | 5.5 |  |  |
| 2001 | 2.1 | 5.3 |  |  |
| 2002 | 3.1 | 3.3 |  |  |
| 2003 | 4.9 | 3.6 |  |  |
| 2004 | 3.3 | 6.4 |  |  |
| 2005 | 4.6 | 5 |  |  |
| 2006 | 5.2 | 4.8 |  |  |
| 2007 | 4.5 | 4.1 |  |  |
| 2008 | 4.7 | 3.8 |  |  |
| 2009 | 2.2 | 2.8 |  |  |
| 2010 | 5 | 5.8 |  |  |
| 2011 | 4.2 | 4 |  |  |
| 2012 | 5.9 | 5.2 |  |  |
| 2013 | 6.1 | 4 |  |  |
| 2014 | 8 | 5.1 |  |  |
| 2015 | 6.7 | 4.6 | 1.4 | 1.1 |
| 2016 | 8.4 | 5.7 | 1.7 | 0 |
| 2017 | 7.5 | 5.3 | 1.8 | 1.4 |
| 2018 | 5.4 | 2.2 | 1.2 | 1.2 |
| 2019 | 6.9 | 6.9 | 1.4 | 0.9 |
| 2020 |  |  | 0.4 | 0.7 |

Rectum

| Years | Rectum (Male) | Rectum (Female) | Rectum (Male) | Rectum (Female) |
| --- | --- | --- | --- | --- |
| 2000 | 7.3 | 5.7 |  |  |
| 2001 | 3.7 | 4.1 |  |  |
| 2002 | 5.6 | 4.7 |  |  |
| 2003 | 1.3 | 3.6 |  |  |
| 2004 | 3.4 | 4.1 |  |  |
| 2005 | 6.1 | 5.2 |  |  |
| 2006 | 2.2 | 5.1 |  |  |
| 2007 | 6 | 6.6 |  |  |
| 2008 | 1.6 | 4.6 |  |  |
| 2009 | 4.6 | 3.4 |  |  |
| 2010 | 5.7 | 2.2 |  |  |
| 2011 | 5.5 | 5.5 |  |  |
| 2012 | 3.6 | 5.1 |  |  |
| 2013 | 4.9 | 5.6 |  |  |
| 2014 | 5.4 | 3.4 |  |  |
| 2015 | 4.4 | 3.8 | 1.4 | 0 |
| 2016 | 4.3 | 2.5 | 1.6 | 0 |
| 2017 | 5.3 | 4.6 | 0.4 | 0 |
| 2018 | 3.5 | 3.8 | 3.7 | 0 |
| 2019 | 1.8 | 3.4 | 1.5 | 1.7 |
| 2020 |  |  | 0.8 | 0.8 |

**Figure 1:** CRC Age-standardised incidence rate for colon and rectum cancers, KCR (2000-2019) and GCR (2015-2020)

a

**Figure 2:** **(a)** Colon cancer (Females) Note: Upper graph: 0 join points; Lower graph: 1 join point

b

c

**Figure 2:** Join point regression for trends in **(b)** Rectum cancer (Males); **(c)** Rectal cancer (Females) Note: Upper graph: 0 join points; Lower graph: 1 join point

**C**

**B**

**A**

**D**

**E**

**F**

**Figure 3:** Kaplan-Meier survival curves showing the survival probability of CRC patients in KCR (A) by Sex; (B) Age at diagnosis; (C) by tumour sub-site; (D) by year of diagnosis; (E) by tumour stage; (F) Overall

**Sensitivity analysis of survival estimates, including all CRC cases retrieved without exclusion of non-histologically confirmed cases**

**Figure 4:** Flow diagram showing CRC cases considered in the different analyses conducted.

GRC, 2015-2020 (n=42)

KCR, 2000-2019 (n=1088)

Total cases retrieved (n=1130)

Total cases for incidence analysis (n=1118)

Excluded atypical primary CRC histology types (n=11) and non-CRCs, anal canal (n=1)

Analysed for incidence rates.

KCR (n=1076)

GCR (n=42)

(n=1131)

Survival analysis

KCR (n= 631)

GCR (n=0)

Exclusions in KCR

Incomplete date of last known vital status (last contact date same as diagnosis date) (n= 311)

Invalid calculation dates (n=134)

Exclusions in GCR

Incomplete date of last known vital status (last contact date same as diagnosis date) = (n=42)

KCR = Kampala Cancer Registry, GCR= Gulu Cancer Registry

Total CRC cases retrieved.

Incidence analysis

Survival analysis

**Table 1:** Survival of CRC patients, KCR (2000-2019)

| **Sub group** | **No. of cases** | **Year 1** | | | **Year 2 & 3** | | | **Year 4 & 5** | | | Median observed survival time (months) |
| --- | --- | --- | --- | --- | --- | --- | --- | --- | --- | --- | --- |
|  | **Alive at start, n** | **Deaths,**  **n (%)** | **LTFU,**  **n (%)** | **OS, 1-year**  **% (95% C.I)** | **Deaths,**  **n (%)** | **LTFU,**  **n (%)** | **OS, 3-year**  **% (95% C.I)** | **Deaths,**  **n (%)** | **LTFU,**  **n (%)** | **OS, 5-year**  **% (95% C.I)** |  |
| Overall | 631 | 205 (32.5) | 315 (49.9) | 42.4 (36.8, 47.9) | 67 (10.6) | 22 (3.5) | 13.1 (9.1, 17.9) | 12 (1.9) | 7 (1.1) | 4.4 (1.9, 8.6) | 11 |
| **By Sex** |  |  |  |  |  |  |  |  |  |  |  |
| Males | 319 | 111 (34.8%) | 151 (47.3) | 41.5 (33.7, 49.0) | 37 (11.6) | 7 (2.2) | 11.6 (6.7, 18.0) | 8 (2.5) | 4 (1.3) | 3.3 (1.0, 8.3) | 9 |
| Females | 312 | 94 (30.1) | 164 (52.6) | 43.3 (35.1, 51.2) | 30 (9.6) | 15 (4.8) | 14.8 (8.8, 22.4) | 4 (1.3) | 3 (1.0) | 6.8 (2.2, 14.9) | 10 |
| **By tumour site** |  |  |  |  |  |  |  |  |  |  |  |
| Colon | 334 | 107 (32.0) | 172 (51.5) | 41.8 (33.9, 49.5) | 34 (10.2) | 9 (2.7) | 13.6 (8.2, 20.4) | 5 (1.5) | 6 (1.8) | 6.0 (2.1, 13.0) | 9 |
| Rectum | 297 | 98 (33.0) | 143 (48.1) | 43.1 (35.1, 50.9) | 33 (11.1) | 13 (4.4) | 12.7 (7.1, 19.8) | 7 (2.4) | 1 (0.3) | 2.9 (0.6, 8.7) | 10 |
| **By stage** |  |  |  |  |  |  |  |  |  |  |  |
| Stage I & II | 53 | 22 (41.5) | 12 (22.6) | 46.8 (31.1, 61.0) | 13 (24.5) | 1 (1.9) | 14.1 (5.4, 26.7) | 5 (9.4) | 0 (0.0) | 0 | 11 |
| Stage III & IV | 73 | 34 (46.6) | 18 (24.7) | 40.1 (27.2, 52.8) | 15 (20.5) | 4 (5.5) | 8.2 (2.4, 18.9) | 0 (0.0) | 2 (2.7) | - | 9 |
| Unknown | 505 | 149 (29.5) | 285 (56.4) | 42.9 (36.0, 49.5) | 39 (7.7) | 17 (3.4) | 14.7 (9.4, 21.2) | 7 (1.4) | 5 (1.0) | 6.8 (3.0, 7.8) | 10 |
| **By age at diagnosis** |  |  |  |  |  |  |  |  |  |  |  |
| <50 years | 250 | 84 (33.6) | 116 (46.4) | 42.8 (34.2, 51.1) | 28 (11.2) | 14 (5.6) | 12.5 (6.7, 20.2) | 6 (2.4) | 1 (0.4) | 2.1 (0.2, 8.9) | 10 |
| 50-69 years | 258 | 78 (30.2) | 134 (51.9) | 44.6 (35.5, 53.3) | 27 (10.5) | 7 (2.7) | 16.2 (9.6, 24.2) | 4 (1.6) | 6 (2.3) | 9.0 (3.8, 17.0) | 11 |
| 70+ years | 123 | 43 (35.0) | 65 (52.8) | 37.3 (24.5, 50.2) | 12 (9.8) | 1 (0.8) | 7.5 (2.0, 18.0) | 2 (1.6) | 0 (0.0) | 0 | 8 |
| **By year of diagnosis** |  |  |  |  |  |  |  |  |  |  |  |
| Year 2000-2010 | 135 | 61 (45.2) | 58 (43.0) | 29.1 (18.6, 40.4) | 8 (5.9) | 3 (2.2) | 12.3 (5.0, 23.0) | 3 (2.2) | 1 (0.7) | 4.9 (1.0, 14.2) | 6 |
| Year 2011-2019 | 496 | 144 (29.0) | 257 (51.8) | 46.1 (39.6, 52.4) | 59 (11.9) | 19 (3.8) | 13.4 (8.8, 18.9) | 9 (1.8) | 6 (1.2) | 4.2 (1.4, 9.5) | 11 |

LTFU: Lost to follow up, OS: Observed survival, C.I: Confidence Interval.

|  | Relative Survival (1-Year) |
| --- | --- |
| Overall | 51.6 |
| Sex Male | 53.8 |
| Sex Female | 48 |
| Tumour site Colon | 52.7 |
| Tumour site Rectum | 49.6 |
| Tumour stage Stage I & II | 54.6 |
| Tumour stage Stage III & IV | 46 |
| Tumour stage Unknown | 53 |
| Age <50 years | 47.9 |
| Age 50-69 years | 52.2 |
| Age 70+ years | 61.2 |
| Year Year 2000-2010 | 39.2 |
| Year Year 2011-2019 | 54.1 |

|  | Relative Survival (3-Year) |
| --- | --- |
| Overall | 14.4 |
| Sex Male | 14.1 |
| Sex Female | 13.7 |
| Tumour site Colon | 14.9 |
| Tumour site Rectum | 13.6 |
| Tumour stage Stage I & II | 16 |
| Tumour stage Stage III & IV | 10.8 |
| Tumour stage Unknown | 15.2 |
| Age <50 years | 10.7 |
| Age 50-69 years | 18.9 |
| Age 70+ years | 13.6 |
| Year Year 2000-2010 | 10 |
| Year Year 2011-2019 | 15.2 |

|  | Relative Survival (5-Year) |
| --- | --- |
| Overall | 4.5 |
| Sex Male | 4.6 |
| Sex Female | 4.2 |
| Tumour site Colon | 6.3 |
| Tumour site Rectum | 2.6 |
| Tumour stage Stage I & II | 0 |
| Tumour stage Stage III & IV | 0 |
| Tumour stage Unknown | 6.9 |
| Age <50 years | 3.2 |
| Age 50-69 years | 8.4 |
| Age 70+ years | 0 |
| Year Year 2000-2009 | 3.2 |
| Year Year 2010-2019 | 5.8 |

**Figure 5:** Relative survival rates for CRC patients in the Kampala Cancer Registry, considering all cases (n=631)
